# Prevention of sick leave at the workplace: design of a cluster-randomized controlled trial of a problem-solving intervention among employees with common mental disorders

**DOI:** 10.1186/s12889-021-11786-6

**Published:** 2021-09-26

**Authors:** E. Björk Brämberg, B. Arapovic-Johansson, U. Bültmann, P. Svedberg, G. Bergström

**Affiliations:** 1 Institute of Environmental Medicine, Unit of Intervention and Implementation Research for Worker Health, Karolinska Institutet, Stockholm, Sweden; 2 University of Groningen, University Medical Center Groningen, Department of Health Sciences, Community & Occupational Medicine, Groningen, The Netherlands; 3 Division of Insurance Medicine, Department of Clinical Neuroscience, Karolinska Institutet, Stockholm, Sweden; 4 Department of Occupational Health Sciences and Psychology, University of Gävle, Gävle, Sweden

**Keywords:** Adjustment disorder, Anxiety disorder, Blue-collar worker, Cluster-randomized controlled trial, Common mental disorder, Depression, Manager, Prevention, Problem solving, Sick leave

## Abstract

**Background:**

Common mental disorders are highly prevalent in the working population, affecting about 1 in 5 persons in the Organisation for Economic Co-operation and Development countries. About 30% of those affected have a first period of sick leave. Despite several attempts to reduce the risk of sick leave among employees with common mental disorders, there is a lack of knowledge about effective, preventive interventions which aim to reduce such risks. This protocol describes the design of a study to evaluate the effectiveness of a problem-solving intervention delivered by first-line managers to employees with common mental disorders on the prevention of sick leave during the 12-month follow-up.

**Methods/design:**

The study applies a two-armed cluster-randomized trial design of a problem-solving intervention conducted in private-sector companies. First-line managers are randomized into intervention- or control groups by computer-generated random numbers, allocation ratio 1:1. Employees are eligible if at risk for future sick leave due to common mental disorders. These are identified by self-reported psychological health measured by the General Health Questionnaire 12-item, cut-off ≥3, or a positive answer to risk of sick leave.

The intervention is based on problem-solving principles. It involves the training of the first-line managers who then deliver the intervention to employees identified at risk of sick leave. First-line managers in the control group receives a lecture. Primary outcome is number of registered days of sick leave due to common mental disorders during the 12-month follow-up. Secondary outcomes are general health, psychological symptoms, work performance, work ability and psychosocial work environment. A process evaluation will examine the intervention’s reach, fidelity, dose delivered, dose received, satisfaction and context. Research assistants managing the screening procedure, outcome assessors and employees are blinded to randomization and allocation.

**Discussion:**

The study includes analyses of the intervention’s effectiveness and an alongside process evaluation. Methodological strengths and limitations, for example the risk of selection bias, attrition and risk of contamination are discussed.

**Trial registration:**

Clinicaltrials.gov NCT04975750 Date of registration: 08/16/2021.

## Background

Common mental disorders (CMD), i.e. depression, anxiety, adjustment disorders and stress-related mental disorders, are highly prevalent in the working population and have been estimated to affect about 1 in 5 persons in countries belonging to the Organisation for Economic Co-operation and Development (OECD) [1, 2]. CMDs have major consequences, affecting individuals and the society at large [3]. CMDs cause individual suffering and pose a risk of social isolation, stigmatization, and long-term sick leave [4]. In the European Union, the estimated annual costs related to mental ill-health were more than 600 billion Euros in 2015, including costs for health care systems, social security programs and indirect costs (i.e. sick leave and impaired work performance) [2].

For employees in general, and specifically for those suffering from CMDs, having paid employment is beneficial for their health [5–8]. Modini et al. identify several individual benefits of employment compared to unemployment. These include increased sense of autonomy, improved self-reported well-being, reduced depression and anxiety symptoms, increased ability to cope with demands, enhanced social status, and unique opportunities for personal development and mental health promotion [5]. The vast majority of individuals with CMDs is employed and about 30% of these employees have a first period of sick leave due to CMDs during adulthood. Around 20–30% of the 30% of employees with a first sick leave spell have recurrent sick leave episodes [9]. Hence, the total burden of sick leave (e.g. individual suffering, isolation, economic loss and societal costs) and the individual benefits of employment emphasize the importance of sick leave prevention.

The current protocol reports the design of a cluster-randomized controlled trial of measures to prevent sick leave. It is based on an effective intervention carried out in the Netherlands by Lexis et al. [10]. The authors evaluated a problem-solving intervention among employees with high risk of sick leave due to depression. The intervention was delivered by specially trained psychologists and was found to prevent future long-term sick leave, defined as more than 28 consecutive days, and to reduce depression [10]. The results indicate the preventive potential of the problem-solving intervention, in particular with regard to long-term sick leave. Given the knowledge that a first period of sick leave can be followed by recurrent spells of sick leave, there is still a need of develop and evaluate interventions which aim to prevent the risk of entering sick leave in the first place.

Several randomized controlled trials evaluating the effectiveness of workplace interventions have been conducted among employees with CMDs or mental health symptoms. Their aim has been to decrease the number of sick leave days [11], prevent long-term sick leave [10], increase return-to-work (RTW) [12], or prevent work disability [13]. Some positive results have been reported, even if the long-term effects of workplace interventions for sustainable RTW are still unclear [11, 12]. One such intervention is a problem-solving intervention based on the principles of cognitive behavioral therapy (CBT) [11]. Evaluations of the effectiveness have shown some positive results in terms of reducing sick leave [14], preventing recurrent sick leave [15], preventing future long-term sick leave, reducing depression [10] and increasing partial RTW [16]. Another workplace intervention is the participatory approach (PA), which is a stepwise intervention involving managers and employees aimed to identify and implement solutions for RTW [17]. The PA has been shown to reduce time until lasting RTW has been achieved among those employees whose baseline measurement indicated an intention to RTW despite having symptoms [18]. In addition, the PA has been evaluated regarding the intervention’s impact on managers’ self-efficacy in preventing sick leave among their employees. It did not, however, result in a reduction of sick-listed employees or sick-leave duration [19].

Workplace interventions at the individual level – irrespective of whether they target employees who are at risk of or already on sick leave – often involve the employee, the employer and a health care or occupational health service professional. Typically, these interventions support the employee in identifying and managing stressors, or in dealing with barriers to RTW, thus placing the responsibility on the individual to adapt to the current situation [20, 21]. There is ample evidence that the psychosocial work environment - such as experiencing fair treatment and having influence over one’s work - is vital for the employees’ mental wellbeing [22, 23]. In the current study, the problem-solving intervention is used as a means to identify and manage adverse psychosocial work environment factors and to enhance the influence over their work situation [12, 22].

Hence, by applying a structured problem-solving process, addressed in stepwise meetings between the first-line manager and their employee, the problem-solving intervention is expected to reduce the risk of sick leave among employees with early signs of CMDs.

The overall aim of this cluster-randomized controlled trial is to evaluate the effectiveness of a problem-solving intervention for the prevention of sick leave among employees with early signs of CMDs. The intervention is delivered by first-line managers and a 30% reduction in sick leave days is expected in the experimental condition compared to the control condition, during the 12-month follow-up. In an alongside process evaluation on the intervention’s core activities, i.e. identification of early signs of CMDs, information to first-line managers about worker health and early signs of CMDs, training of first-line managers in problem-solving conversation and communication, we will:

- evaluate to what extent it was possible for the first-line managers to adhere to the intervention’s protocol,
- investigate the association between the intervention’s core activities and number of sick leave days,
- identify the facilitators or and barriers to the intervention among first-line managers and employees and,
- explore the first-line manager’s organizational resources supporting their general managerial work.

The process evaluation will provide in-depth knowledge and insights into the intervention’s mechanisms and will make it possible to identify determinants of an effective intervention implementation and of change mechanisms. The study will be conducted in private sector companies among first-line managers and blue-collar workers. The outcomes will be evaluated at both cluster- and individual participant level.

## Methods/design

**Fig. 1:**
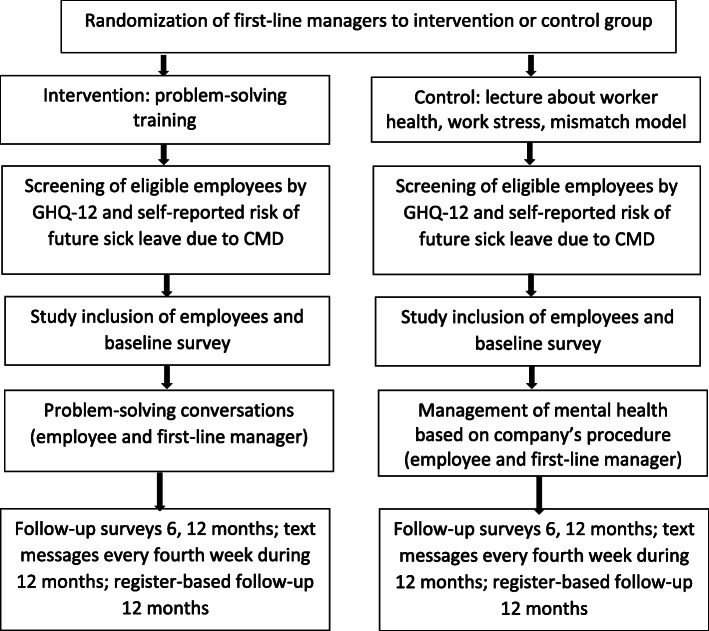
Flowchart and overview of the trial

The study applies a two-armed cluster-randomized controlled trial design of a problem-solving intervention to prevent sick leave among occupationally active, blue-collar employees with CMDs (Fig. 1). The description of the study design follows the CONSORT statement for randomized controlled trials, with extension to cluster randomized controlled trials [24, 25]. The trial involves a psychologist training of first-line managers to deliver the intervention, developed in the Netherlands [10] and adapted to the Swedish context. First-line managers are commonly the first ones to identify employees with a reduced work performance or who are at risk for sick leave due to health complaints. This is also in line with the PA intervention with a stepwise protocol for meetings between manager and employee [17].

The study will take place at several private companies. Randomization will take place at the level of the first-line managers, i.e. those with personnel responsibility. The employees will follow their first-line manager’s randomization. The employees will have access to the occupational health services (funded by each company) or primary health care (publicly funded), if the screening identifies severe conditions that necessitate care by medical professionals.

**Fig. 2:**
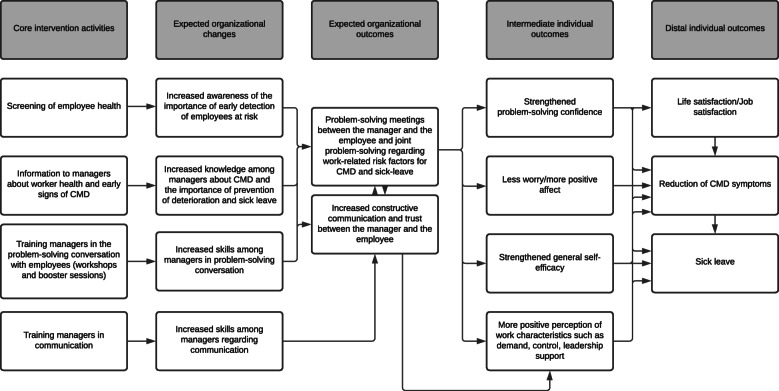
The problem-solving intervention’s program theory with the intervention’s core activities, expected changes and outcomes on organizational and individual levels

The trial’s program theory with expected changes and outcomes is presented in a logic model (Fig. 2).

### Context

In Sweden, individuals above the age of 16 who cannot work due to disease or injury are entitled to full or part-time sick leave (25, 50, 75 or 100%). Those who are gainfully employed receive economic compensation from their employer for the first 14 days, except for one qualification day. Thereafter, the Swedish Social Insurance Agency (SSIA) grants the benefits. The Swedish social insurance system is mainly tax funded. To receive economic compensation for sick leave, a sickness certificate issued by a physician is needed from day 8.

### Participants

#### Recruitment of first-line managers

Information about the study’s aim and design is provided to the steering groups at each company by the principal investigator (EBB).

##### Inclusion criteria

Eligible first-line managers are included if they meet the following criteria:

- ≥1-year work experience as a first-line manager
- Working hours ≥50% of fulltime
- Understand written and spoken Swedish

##### Exclusion criteria

- Planned long-term absence during the coming year (for example parental leave, new job, retirement)

#### Recruitment of employees

To be eligible for participation an employee must be at risk for future sick leave due to CMDs. Employees at risk are identified by a web-based screening questionnaire, administrated by a research assistant to all employees whose first-line managers have been included and randomized. The following health and sick leave measures will be screened by the web-based questionnaire;

Self-reported psychological health is measured by the validated General Health Questionnaire (GHQ) 12-item, Swedish version [26]. The items include aspects reflecting different symptoms primarily related to depression but also general anxiety symptoms, for example stress, and further ability to perform daily activities and coping with everyday problems. Six items are positively worded and six items negatively. The time period assessed is the past few weeks. Every item has a four-category response format, namely “better than usual”, “same as usual”, “less than usual” and “much less than usual” [26, 27]. The traditional scoring method (all items are coded 0–0–1-1) will be used. Individuals scoring ≥3 are categorized as at risk for sick leave due to CMDs. This cut-off has previously been used in the Swedish Public Health Agency’s national surveys [28].

Sick leave risk is measured by a single question, developed by the research group: “About your health – do you think you will receive sick leave benefits because of stress, anxiety or depression in the coming 12 months?”. The response format is a 4-point scale, ranging from “Yes, most likely”, “yes, quite likely”, “I’m not sure”, “no, probably not”. The employee’s own beliefs and/or expectations about future sick leave have been shown to predict sick leave [4, 29].

#### Eligibility criteria

##### Inclusion criteria

Employees will be included if they meet the following criteria:

- women and men, employed in the private sector, aged 18–59 years,
- scoring with a cut-off ≥3 points on the GHQ-12, or a positive answer (i.e. “yes, most likely”, “yes, quite likely”) on the question on sick leave risk,
- understand written and spoken Swedish.

##### Exclusion criteria

- Ongoing sick leave (full- or part-time), leave of absence, pregnancy,
- sick leave ≥14 calendar days during the last 3 months due to CMD
- exposed to workplace bullying by the first-line manager
- planned long-term absence during the coming year (for example parental leave, new job, retirement).

The research assistant screens the incoming answers on the web-based screening questionnaire for inclusion and exclusion criteria. Those who meet the inclusion criteria are informed by the research assistant (via e-mail or a telephone call) about their screening result and are recommended to talk to their first-line managers. Eligible employees follow the randomization of their first-line manager. The research assistant is blinded to group allocation.

### Randomization and blinding

The current study is designed as a two-armed cluster-randomized controlled trial. The randomization of participants is computer-generated, stratified by company, and takes place at the level of first-line managers. They are allocated at the individual level to intervention- or control group by means of computer-generated random numbers with allocation ratio 1:1. The random allocation sequence is generated by an independent statistician. The first-line managers will be enrolled and informed about their allocation by a research assistant.

To avoid contamination, the first-line managers in the intervention arm are instructed to talk and discuss the intervention only with each other and the project team. The first-line managers are not blinded to allocation since they will know about their participation in the problem-solving training (intervention) or the workshop (control condition).

Eligible employees follow the randomization of their first-line manager and are randomized before providing informed consent. All employees, irrespective of whether they are in the intervention- or control group, receive the same information about the study. The information contains details about randomization but not about the content of the two arms, i.e. the employees are blinded for their group allocation. Research assistants, blinded to the first-line managers’ and employees’ randomization, will manage the screening procedure and the outcome assessments (i.e. administration of baseline, follow-up questionnaires and text messages). The statistical analyses will be performed by an independent statistician who will be blinded for the participants’ randomization (both first-line managers and employee).

### The problem-solving intervention

The intervention is based on a problem-solving technique developed by Nezu et al. [30]. The intervention starts with the training of the first-line managers in the problem-solving intervention by a licensed psychologist (Fig. 2). Thereafter, the first-line managers apply the problem-solving conversation in their meetings with employees at risk of future sick leave due to CMDs. The intervention focuses on the individual employee and his/her work situation and work-private life balance and follows the steps described below. The intervention is carried out between the first-line manager and the employee in 2–5 planned meetings (30–45 min) over a time period of 3 months.

Step 1: Inventory and prioritization of problems by the first-line manager and the employee

In the first step, possible problems related to work are identified and ranked. By establishing a trustful communication, the employee is encouraged to describe his/her work and the background to any problems. The employee’s private life and opportunities for time off work and recovery are examined – if they are relevant to the problem and the employee is willing to share the information. All problems (and opportunities) in relation to work, including possible interference between work and private life, are discussed. A preliminary problem list is compiled and the problems are ranked. Next, the ranked problems are described and defined (including the separation of facts and assumptions, and identification of known barriers) and realistic goals are set. The employee is encouraged to reflect on this prioritization and possible solutions before the next meeting.

Step 2: Brainstorming about options and solutions by the first-line manager and the employee

The purpose of the second step is to brainstorm about as many different options and solutions as possible. These options and solutions are then discussed and evaluated with regard to the following:

- will the options and solutions help to solve the defined problem in accordance with the set goals?
- can the solutions realistically be implemented?
- what are the advantages and the disadvantages of the brainstormed solutions?
- what are the short- and long-term consequences for the employee and other stakeholders, such as co-workers, organization?

The employee is encouraged to reflect on the options and solutions before the next meeting.

Step 3: Formulation of an action plan by the first-line manager and the employee

The aim of third step is to reach an agreement about which solution/−s are to be implemented and to develop an action plan. The plan should describe in detail how the solutions will be implemented; who is responsible for the implementation; when the agreed solutions will be implemented; and when the action plan will be evaluated. At least one solution should preferably start soon after the action plan has been agreed upon and be followed-up shortly. Larger-scale solutions should be divided into several steps. The employee and the first-line manager can, if necessary, involve the human resources department, occupational health services etc. Obstacles and alternative plans should be discussed if the action plan does not work out as intended. The meeting should result in a written action plan, signed by the first-line manager and the employee.

Step 4: Evaluation and follow-up by the first-line manager and the employee

In the fourth step, the action plan is followed-up and evaluated. One to three follow-up sessions (depending on the problem(s)) are recommended, preferably during the first 12 weeks after the last problem-solving meeting, however the number of sessions can be adapted to the employee’s needs. If the action plan has had a positive effect on the defined problem, the follow-up meeting is an opportunity to support and encourage the employee in what has been done. If the action plan did not work or if new problems have occurred, it is an opportunity to discuss the reasons and go back to the problem-solving process or initiate other contacts (occupational health services, human resources department, etc.) if additional support is needed.

#### Training of first-line managers in the intervention group

The first-line managers take part in a 1 ½ days training course. The training course is provided by a licensed psychologist (BJ) with extensive experience of problem-solving therapy, manager coaching and supervising health care professionals, and the principal investigator (EBB) who also has a thorough knowledge of problem-solving interventions.

The training is based upon interactive and didactic principles, as recommended by Forsetlund et al. [31]. The training includes didactic features (lectures, reading about problem-solving) and participative sessions that provide the opportunity to practice skills (role-play, observations of others role-play, discussions). The first-line managers receive a manual and worksheets for the intervention [14, 32]. In the current project the manual and worksheets have been adapted for use by first-line managers, because they are not expected to have professional training in e.g. communication skills and/or identifying signs of depressive symptoms. The first-line managers will be trained in conversation and communication with focus on: general communication skills, i.e. to listen actively and attentively (use eye contact with the employee, nod, show that you are listening); encouraging the employee to talk and summarize and check your understanding of what has been said; observing and describing the employee’s feelings without judgment [33]. In addition, the first-line managers receive information about validation of communication, which is about communicating an understanding of the other person’s feelings and thoughts [34]. This form of communication does not necessarily mean agreement; it is rather used as a means to acknowledge and legitimize the other person’s experience, which in turn may increase the likelihood that he or she will feel understood. This may result in building trust, reducing emotional intensity and de-escalating possible conflict [35]. We understand some degree of validation to be a beneficial aspect of any type of successful communication, and especially with employees who are at risk of CMDs. These employees may present with some level of emotional vulnerability. Validating communication on the part of the first-line manager may prevent adding further to this vulnerability. If the communication is dis-validating it could confound the effects of the problem-solving. This part of the training is therefore seen as a prerequisite for successful problem-solving.

Between the first and second training sessions, the managers are expected to complete one or two learning assignments. Opportunities and difficulties regarding using the steps of the problem-solving procedure or other aspects of their meetings with the employee are discussed. Common difficulties encountered in the course of the problem-solving intervention, such as defining problems and brainstorming about solutions [36], will be specifically addressed. In addition, the first-line managers are encouraged to use problem-solving whenever possible.

In addition to the training, the first-line managers will be invited to two web-based booster sessions in the first 8 weeks after the training. These sessions include supervision and feedback about the first-line managers’ use of the problem-solving intervention and are based on the first-line managers’ reported cases and/or difficulties. The sessions are led by the licensed psychologist (BJ) and the principal investigator (EBB).

#### Training of the first-line managers in the control group

The first-line managers randomized to the control group will receive a 3-h lecture which includes a brief overview of worker health, work stress and the mismatch model [37]. The information includes protective and risk factors in the work environment in relation to occupational stress and CMDs. Basic information about the importance of self-efficacy for mental health is given, and different ways of interacting with and supporting employees with CMDs are reflected upon. The lecture also includes advice about communication, with focus on general communication skills: listen actively and attentively; encouraging the employee to talk; summarizing and checking your understanding of what has been said; observing and describing the employee’s feelings without judgment [33].

### Measurements

Data will be collected from registers, self-reported questionnaires, text messages and qualitative interviews. Register data on sick leave will be collected from the employer’s register, and the Micro Data for the Analysis of Social Insurance register (MiDAS) provided by the SSIA. Register data on employee characteristics will be collected from the Longitudinal integrated database for health insurance and labor market studies (LISA) [38]. Web-based questionnaires will be administered at baseline and at 6- and 12-month follow-up. Text messages will be sent every fourth week to the employees during a 12-month period. A 36-month follow-up of sick leave based on register data is planned.

### Primary outcome

#### Sick leave

The primary outcome is the total number of days of sick leave due to CMDs during the 12-month follow-up. Data will be collected for individuals from the participating companies’ (employers’) registers (covering the first 14 calendar days of sick leave) and from the MiDAS register (sick leave ≥15 days) covering part-time (defined as 25, 50 or 75% of full-time) and full-time. Sick leave due to diagnoses other than CMDs will also be included. Data will be collected from baseline until the 12-month follow-up and for the 24 months preceding baseline. The primary outcome is based on data from the employers’ and MiDAS registers, limiting missing values during follow-up.

### Secondary outcomes

Data on the secondary outcomes will be collected by means of self-reported questionnaires and text messages.

#### General health and psychological symptoms

Self-rated general health is measured with a single item from the Short-Form Health Survey with 5-point response anchors, from (1) “excellent” to (5) “bad” [39]. The presence and severity of depression (7 items) and anxiety (7 items) are assessed by the validated Hospital Anxiety and Depression Scale, which is a 14-item self-reported questionnaire [40, 41]. The response format is a 4-point scale, ranging from 0 (not at all) to 3 (nearly all the time), with higher scores indicating higher levels of depression and anxiety. Self-reported exhaustion will be assessed by four items of the self-rated exhaustion disorder (s-ED) scale [42], that has demonstrated construct and predictive validity. The response format is yes/no [42]. Work stress is measured by a single item with a 5-point response scale, ranging from (1) “not at all” to (5) “very much” [43], administered as a text message.

#### Work performance impairment and work ability

Work performance impairment will be evaluated by two items administered as text messages: one item about impairment of work performance due to health problems (presenteeism), and one item about work environment problems. These items have been developed, modified and validated [44] and were inspired by the Work Productivity Activity Impairment questionnaire [45]. The first item, impairment of work performance due to health problems, is assessed by the following question: “During the past four weeks, how much did your health problems affect your performance while you were working?”. The respondents are asked to rate how their health-problems affect their work performance on a scale ranging from 0 indicating that health problems had no effect on their work, to 10, indicating that health problems hindered them from working at all. The second item about work-environment problems is measured by the question: “During the past four weeks, how much did work environment problems affect your performance while you were working?”. The response format is a scale ranging from 0 to 10, with higher scores indicating that work environment problems prevented respondents from working. The items have been tested in a Swedish setting and shown reliable psychometric properties (test-retest ICCs with 95% CIs for health related and work-environment production loss, respectively: 0.90, (CI; 0.74–0.98); 0.91, (CI; 0.79–0.98)) [46].

Work ability will be measured by three items of the Work Ability Index [47]. Two items refer to perceived work ability in relation to the physical and mental demands of the work. The response format is a five-point scale, ranging from “very bad” to “excellent”. The third item assesses the employee’s own beliefs about his/her work ability: “Do you believe that – from the standpoint of your health – you will be able to do your current job two years from now?” with response format “unlikely”, “not certain”, “relatively certain” [47].

#### Psychosocial work environment

The Copenhagen Psychosocial Questionnaire (COPSOQ) III, Swedish standard version [48], is used for assessing *demands at work* by quantitative and emotional demands. *Interpersonal relations and leadership* are assessed by recognition and quality of leadership; *work-individual interface* is measured by commitment to the workplace and work-life conflict. These scales are measured at baseline and at 6- and 12-month follow-up. The response format is a Likert scale with five levels (0, 25, 50, 75, 100), with higher scores indicating higher work demands, better interpersonal relations and leadership, and for the work-individual interface, higher scores indicate better commitment to the workplace and worse work-life conflict.

The impact of private life on work is measured by the single item: “Do the demands of your family or spouse/partner interfere with your work-related activities?” from the General Nordic Questionnaire [49, 50] with the response anchors “Very seldom or never” to “Very often or always”.

#### Intermediate outcomes

General self-efficacy measures the employee’s belief in his/her ability to cope with the current situation, to mobilize motivation and act upon demands in different situations. It is positively correlated with mental work capacity. In this study, general self-efficacy is included to clarify how the intervention is working and is measured by a Swedish-validated version of the General self-efficacy scale [51], at baseline and at 6- and 12-month follow-up. Responses are given on a four-point Likert scale ranging from (1) “not at all true” to (4) “exactly true”. A validated single-item measure of self-efficacy is also used: “I am confident in my ability to solve problems that I might face in life”. The response format is a ten-point scale from (1) “totally disagree” to (10) “totally agree” [52].

In addition, problem-solving might lead to a better perception of influence at work, and manager support, measured by COPSOQ III subscales Influence at work, and Social support from supervisor [48]. Influence at work is assessed by four questions of Influence at work scale (e.g. Do you have any influence over what you do at work?). Manager support is assessed by two questions of the social support from supervisor scale (e.g., How often is your immediate superior willing to listen to your problems a work, if needed?).

#### Prognostic factors

A variety of prognostic factors for the risk of future sick leave due to CMDs will be measured. Employees’ personal characteristics (e.g. age, gender, marital status, educational level, profession) and workplace characteristics (e.g. sector) will be collected from the baseline questionnaire and the LISA and MiDAS registers.

Employees’ psychosocial safety climate (PSC) will be measured at baseline. PSC focuses on the senior management’s role in creating a healthy work environment and refers to the employees’ perceptions of the organization’s psychosocial health climate, e.g. guidelines, practices and procedures used to establish and maintain a psychosocial safe and sound work environment [53]. Further, the concept reveals whether the senior management supports stress prevention at work. The four-item version of the valid and reliable Psychosocial Safety Climate [54] will be used, using a 5-point scale ranging from (1) “strongly disagree” to (5) “strongly agree”. Average scores > 12 indicate good occupational safety and health [55].

Information about workplace adjustments (e.g. changes in work tasks, working hours, change of department) and co-interventions (e.g. psychological counselling, and if so, number of sessions, use of antidepressants, general health care consumption, contact with the occupational health service) will be collected from the employees at baseline and at 6- and 12- month follow-up.

The first-line managers’ personal characteristics (e.g. age, gender, marital status, education) and workplace characteristics (e.g. sector, profession, number of employees, leadership network, leadership training) will be collected. In addition, the first-line managers’ self-efficacy (single item), problem-solving confidence [56], leadership support or supervision, and perceived ability to support employees with CMD, will be measured at baseline and after 12 months.

### Process evaluation

A process evaluation will be conducted alongside the cluster-randomized controlled trial, as recommended by Moore et al. [57]. The process evaluation is based on the theoretical framework by Linnan and Steckler [58] and the Consolidated Framework for Implementation Research (CFIR) [59]. The process evaluation will examine the study’s core intervention activities (i.e. identification of early signs of CMDs, information to first-line managers about worker health and early signs of CMDs, training of first-line managers in problem-solving conversation and communication) with regard to 1) reach, 2) fidelity, 3) dose delivered and dose received, 4) satisfaction and context. Process-evaluation data will be collected at employee- and first-line manager-level, at baseline and during and post-intervention, by means use of self-reported questionnaires and semi-structured interviews.

*Reach* is measured at employee- and first-line manager-level. For employees, reach is defined and measured as the proportion of all employees eligible for inclusion who agree to participate in the study. The first-line managers attendance in the training and booster sessions will be measured by attendance and satisfaction.

*Fidelity* is about adherence to the study protocol and intervention delivery. It is assessed as by whether, and to what extent, it was possible for the first-line manager to deliver the intervention as intended. The first-line managers will provide data on their adherence to the manual for each of their employees during the intervention period.

*Dose delivered* is operationalized as number of times the first-line manager meets with the employee including number of follow-up sessions, as face-to-face meetings or telephone follow-ups, and the content of the meetings and follow-ups. *Dose received* is assessed by to what extent the employee receives the problem-solving intervention, in terms of numbers of meetings and follow-ups with the first-line manager. The employees are asked to rate the quality of the meetings with their first-line manager.

*Satisfaction and context* among first-line managers and employees are measured with self-reported questionnaires (first-line managers) and qualitative semi-structured interviews (first-line managers and employees). Satisfaction is defined as how participation in the intervention is perceived, for example the perceived benefits of taking part, or the resources needed for the delivery of the intervention. Context is operationalized as the perceived barriers and facilitators which influence the implementation of the intervention. First-line managers will be asked to rate the resources available for them for delivering the intervention, e g. time, managerial support. The interview guides will be developed by the research team. Questions specifically related to the context will be based on the CFIR guide to enable a systematic identification of barriers to and facilitators of the intervention [59, 60].

### Data analysis

Multilevel analyses for cluster-randomized trials will be conducted. To investigate the effectiveness of the primary and secondary outcomes, we will perform intention-to-treat analyses using linear and generalized models taking the multilevel nature into account. The primary outcome will be analyzed using generalized estimating equations with independent correlation structure and robust variance estimation.

The primary outcome (number of days of sick leave due to CMDs during the 12-month follow-up) will be analyzed from baseline during the 12-month follow-up. The parameter of interest will be the group allocation. The analysis will, for example, be conducted using regression-based methods that allow the intra group correlation induced by the clustered randomization design. Group-based trajectory models will be used to map the developmental course of symptoms, and to assess the heterogeneity in response to clinical interventions [61].

A longitudinal analysis of days of sick leave and of secondary outcomes can also be conducted, given the intra-person longitudinal nature of the data. This can be done at day, week, month or quarter level, depending on the granulation of the data and the nature of the outcome of interest.

Semi-structured interviews collected as process-evaluation data of perceived barriers to and facilitators of the intervention will be analyzed by qualitative content analysis [62] or thematic analysis [63].

#### Statistical power

By sampling 38 clusters (19 clusters in the intervention arm and control arm respectively) with an average of three employees in each cluster, the study population is estimated to be a total of 114, with 57 employees in the intervention group and the control group respectively. With these numbers it will be possible to achieve about 80% power to detect a difference of 30% in number of days of sick leave during the 12-month follow-up, assuming that the mean number of days in the control group is 108 [14]. The standard deviation of sick leave days was 60 days [10]. The intra-cluster correlation was set to 0.015 and a significance level of 0.05, two-sided test.

## Discussion

This paper describes the design of a cluster-randomized controlled trial of a problem-solving intervention for employees at risk of future sick leave due to CMDs. It will be conducted in the workplace, with first-line managers delivering the intervention or a control condition. The primary aim is to evaluate the intervention’s effectiveness to prevent sick leave due to CMDs. The secondary aims are to assess the effects of the intervention on general health, psychological symptoms, work performance and work ability, the psychosocial work environment and the psychosocial safety climate. To the best of the authors’ knowledge, this study is the first to evaluate the problem-solving intervention as a preventive intervention including first-line managers.

The present study employs an intervention starting with training of the first-line managers, and thereafter, the first-line managers use the problem-solving intervention in meetings with their employees who are at risk of future sick leave. Previous studies point to the potential of problem-solving confidence and self-efficacy mediating outcomes such as positive affect, job- and life satisfaction [64], which in turn, are associated with mental health and subjective wellbeing [65, 66]. The problem-solving intervention used in the current study is a short intervention, consisting of 2 to 5 sessions. By means of a thorough, mixed-method process evaluation based on a systematic approached using complementary theoretical frameworks [58, 59], data are collected at employee- and first-line manager levels, and at different timepoints, i.e. before, during and post intervention. The added value of the process evaluation is the link with the findings from the effectiveness evaluation – i.e. to connect the findings of both evaluations. Hence, the process evaluation will enable us to gain in-depth knowledge and insights into the intervention’s mechanisms. It will also make it possible to identify determinants of effective intervention implementation and change mechanisms [67, 68] linked with the effect evaluation.

Previous research indicates that employees with CMDs or mental health symptoms should be given the opportunity to plan and pace their own work [69]. In most cases, support to implement work adjustments from, for example, the first-line manager is needed, because he/she is responsible for leading and allocating the work. In 2015, new provisions about the organizational and social work environment were introduced in Sweden [70]. The new provisions place increased responsibility on the employer to assess physical and psychosocial risks at work and to implement measures to identify, manage and correct potential and real risks. The problem is that first-line management does not always know how to go about this and the provisions do not give guidelines. The problem-solving methodology described in this protocol may be used as a tool for these “corrective measures” with preventive purposes.

The intervention raises ethical issues about personal integrity in relation to employees’ private and working lives and the possible negative consequences of revealing such information to the first-line manager. The employee participating in the current study has the right to choose which matters and/or problems to discuss with the first-line manager and to maintain private life integrity. This is stated in the written information that the employee receives before giving consent to participation. When taking part in the meetings with the first-line manager, the employee is instructed to talk about matters that – from the employee’s perspective - affect his/her work and work tasks. If successful, the problem-solving process may also be applied to the intersection between private life and working life.

### Strengths and limitations

The current study has some important strengths. To start with, a cluster-randomized controlled trial design will be used when evaluating the effectiveness of the problem-solving intervention on the primary outcome, i.e. register-based days of sick leave. The trial will be conducted at several workplaces; these are settings in which there are limited opportunities to conduct a rigorous randomized controlled trial. The cluster-design will allow the first-line managers to provide either the problem-solving intervention or the control condition. The first-line managers in the intervention group will also be told not discuss the intervention with their colleagues outside the intervention group. An additional strength is the use of register data for measuring the primary outcome. The data from the employer’s register and MiDAS will provide objective data about sick leave from day one, and we expect no missing data.

Another strength is the recruitment procedure. We will use a screening procedure to identify employees with early signs of CMD and at risk of future sick leave due to CMDs. Research assistants will screen the incoming answers and administer the baseline measurement to those meeting the inclusion criteria. The assistants have no previous knowledge about the employees and are blinded to group allocation. By using this procedure, we can reduce the risk of selection bias.

We must also mention some limitations. The screening of employees’ risk of future sick leave due to CMDs will be carried out among all employees whose first-line manager participates in the study. There is a potential risk that only employees who have confidence in and a trustful relationship with their first-line manager will respond to the questionnaire, implying that largely motivated employees or employees who only have minor problems will be included in the study. This may affect the generalizability of the study. However, participation is voluntary for both first-line managers and employees. Another limitation is the data collection, with self-reported questionnaires administered at baseline and at 6- and 12-month follow-up, and text messages sent every fourth week during the 12-month follow-up. We cannot rule out the risks of recall bias. However, we suggest that the response burden is appropriate.

### Impact of results

Finding effective ways to prevent or at least reduce the risk of sick leave due to CMDs and using replicable interventions will help employees’ mental health and economic situation and offer benefits in the workplace and in society at large.

There is ample evidence that the psychosocial work environment is vital for the mental well-being of workers, yet many interventions fail to address the psychosocial work environment. Thus, there is a lack of knowledge about workplace interventions which target both the individual employee and organizational change by promoting a good psychosocial work environment. In line with this, the present study will contribute to the area by developing knowledge from a preventive perspective and focusing on employees, first-line managers and their workplaces. This cluster-randomized controlled trial will test the effectiveness of an intervention based on problem-solving principles which aims to prevent the risk of sick leave due to CMDs.

## Data Availability

The current article describes a study protocol, and therefore data sharing is not applicable as no datasets have yet been generated or analyzed.

## Abbreviations

CBT: Cognitive Behavioral Therapy
CFIR: Consolidated Framework for Implementation Research
CMD: Common Mental Disorders
COPSOQ: Copenhagen Psychosocial Questionnaire
GHQ: General Health Questionnaire
LISA: Longitudinal integrated database for health insurance and labor market studies
MiDAS: Micro Data for the Analysis of Social Insurance
PSC: Psychosocial Safety Climate
RTW: Return to Work
OECD: Organisation for Economic Co-operation and Development
s-ED: Self-rated exhaustion Disorder
SSIA: Swedish Social Insurance Agency
